# Short-Chain Fatty Acids (Except Hexanoic Acid) Lower NF-kB Transactivation, Which Rescues Inflammation-Induced Decreased Apolipoprotein A-I Transcription in HepG2 Cells

**DOI:** 10.3390/ijms21145088

**Published:** 2020-07-18

**Authors:** Jehad Z. Tayyeb, Herman E. Popeijus, Ronald P. Mensink, Maurice C. J. M. Konings, Fatma B. A. Mokhtar, Jogchum Plat

**Affiliations:** 1 Department of Nutrition and Movement Sciences, NUTRIM School for Nutrition and Translational Research in Metabolism, Maastricht University, 6229 ET Maastricht, The Netherlands; h.popeijus@maastrichtuniversity.nl (H.E.P.); r.mensink@maastrichtuniversity.nl (R.P.M.); maurice.konings@maastrichtuniversity.nl (M.C.J.M.K.); fatma.mokhtar@maastrichtuniversity.nl (F.B.A.M.); j.plat@maastrichtuniversity.nl (J.P.); 2 Department of Clinical Biochemistry, Faculty of Medicine, University of Jeddah, Jeddah 23218, Saudi Arabia

**Keywords:** ApoA-I, inflammation, NF-κB, PPARα, SCFAs

## Abstract

Concentrations of apolipoprotein A-I (ApoA-I) decrease during inflammation, which may lead to dysfunctional ApoA-I-poor high-density lipoprotein (HDL) particles, and as such, elevate cardiovascular risk. Therefore, rescuing ApoA-I concentrations, especially during inflammation, seems beneficial. Recently, short-chain fatty acids (SCFAs) have received more attention as a strategy in reversing atherosclerosis. We here evaluated the effects of SCFAs on inflammatory pathways in relation to ApoA-I transcription. SCFAs dose–response studies were performed in the presence and absence of inflammatory cytokines. ApoA-I and interleukin 8 (IL-8) mRNA expression were analyzed using qPCR and ELISA, respectively. To study underlying mechanisms, nuclear factor kappa B (NF-κB) transactivation and changes in mRNA expressions of the genes targets of bromodomain and extra-terminal (BET) inhibition, peroxisome proliferator-activated receptor-alpha (PPARα) transactivation and activator protein 1 (AP-1) pathway were analyzed. SCFAs (except hexanoic acid) increased ApoA-I mRNA transcription in both normal and inflammatory conditions and lowered IL-8 mRNA expression. This anti-inflammatory effect of SCFAs was confirmed by inhibition of NF-κB transactivation. Moreover, butyric acid increased carnitine palmitoyltransferase 1 (CPT1), PPARα target gene, mRNA transcription in both conditions, and there was a negative correlation between CPT1 and NF-κB. Therefore, PPARα transactivation is probably involved in the anti-inflammatory effects of SCFAs, which rescues ApoA-I transcription. In conclusion, propionate, butyrate and valerate elicit anti-inflammatory effects which might rescue ApoA-I transcription in inflammatory conditions via PPARα transactivation mediated NF-κB inhibition.

## 1. Introduction

Inflammation has clear effects on lipid and lipoprotein metabolism, which contributes to the association between inflammation and increased cardiovascular risk as seen for example in metabolic syndrome [1]. It is known that inflammation strongly reduces high-density lipoprotein (HDL) cholesterol and particle concentrations and, more importantly, introduces the formation of dysfunctional HDL particles which consequently leads to impaired reverse cholesterol transport [2]. Therefore, elevation of HDL particle concentration and more particularly its functionality, especially in inflamed conditions, might help to prevent the development of atherosclerosis and cardiovascular diseases (CVD) [3]. The potential anti-atherogenic effects of HDL have been linked to its main functional and structural protein, apolipoprotein A-I (ApoA-I) [4]. Amongst others, ApoA-I is the acceptor for ATP-binding cassette transporter (ABCA1)-mediated cholesterol efflux and as such, regulates cholesterol efflux capacity [5]. In addition, ApoA-I has many other functional and cardioprotective effects such as blunting inflammation [6] and lowering coagulant activity [7,8]. This explains the cross-sectional associations between ApoA-I and lower CVD risk. Lately, short chain fatty acids (SCFAs) have received more attention as they may be an attractive strategy in reversing the pathophysiology of metabolic disorders such as chronic inflammation and atherosclerosis [9]. SCFAs are either absorbed and utilized by gut epithelial cells or transported directly to the liver via the portal vein [10]. We have shown earlier that exposure to SCFAs elevates in vitro ApoA-I production in HepG2 cells [11]. Moreover, Bartolomaeus and coworkers have recently shown that propionate (C3) supplementation reduced atherosclerosis in experimental animal models [12]. This is in line with earlier observations suggesting a protective role for SCFAs in CVD development and inflammatory diseases [13]. Furthermore, SCFAs can inhibit the proliferation and activation of T-cells and block the adhesion of antigen presenting cells in obesity-associated systemic inflammation [14]. Given these effects of SCFAs on inflammation, here we evaluated the effects of SCFAs on the inflammatory pathways in relation to ApoA-I transcription in inflamed HepG2 cells and attempted to unravel the mechanism underlying these effects.

## 2. Results

### 2.1. Effects of SCFAs on ApoA-I mRNA Expression in Normal and Inflammatory Conditions

**Figure 1:**
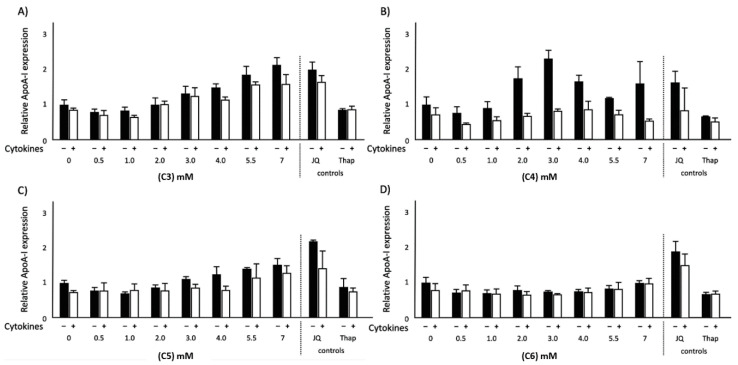
Relative apolipoprotein A-I (ApoA-I) mRNA expression in HepG2 cells treated with different concentrations of short chain fatty acids (SCFAs), JQ1(+) (3 μM) or thapsigargin (Thap) (0.01 μM). Six biological (12 technical) replicates were performed for every condition. (**A**) Increasing C3 concentrations showed a significant increase in ApoA-I mRNA expression in both normal and inflammatory conditions (*p* < 0.05). (**B**) Increasing C4 concentrations showed a significant increase in ApoA-I mRNA expression in both normal and inflammatory conditions (*p* < 0.05). (**C**) Increasing C5 concentrations showed a significant increase in ApoA-I mRNA expression in both normal and inflammatory conditions (*p* < 0.05). (**D**) Increasing C6 concentrations did not show any significant effects in ApoA-I mRNA expression in both normal and inflammatory conditions. The positive control JQ1(+) and the negative control Thap respectively increased and decreased ApoA-I mRNA expression (*p* < 0.001) in both normal and inflammatory conditions. All results are presented as the mean, while error bars indicate standard deviations. Data were normalized against the expression observed in the control condition, which was arbitrarily set at 1. Linear regression for SCFA dose–response effects was performed except for C4, where a quadratic polynomial regression was performed to evaluate the dose–response effects. Changes were considered significant when the regression coefficients were significantly different from zero (*p* < 0.05).

ApoA-I mRNA expression in HepG2 cells was lower under inflammatory conditions as compared to normal conditions (Figure 1). In agreement with our previous studies [15], the positive control JQ1(+) and the negative control thapsigargin (Thap) respectively increased and decreased ApoA-I mRNA expression (both *p* < 0.001). Also, under inflammatory conditions, JQ1(+) significantly (*p* < 0.001) increased ApoA-I gene expression, whereas Thap even further decreased ApoA-1 gene expression (*p* < 0.001) compared with the normal condition (Figure 1). For the SCFAs, we observed that both under normal and inflammatory conditions, ApoA-I mRNA expression dose-dependently increased after C3, butyric acid (C4) and valeric acid (C5) treatment (*p* < 0.05), whereas hexanoic acid (C6) did not change ApoA-I mRNA expression. Interestingly, all SCFAs (except C6) were able to rescue the reduced levels of ApoA-I mRNA under the inflammatory condition. Both C3 and C5 fully rescued ApoA-I mRNA expression at 5.5 mM, and even increased ApoA-I mRNA expression at 7 mM, while C4 partly rescued ApoA-I mRNA expression up to 80% at 4 mM (Figure 1).

### 2.2. Effects of C4 on KEAP1 and CPT1 mRNA Expression and NF-κB Transactivation in Normal and Inflammatory Conditions

**Figure 2:**
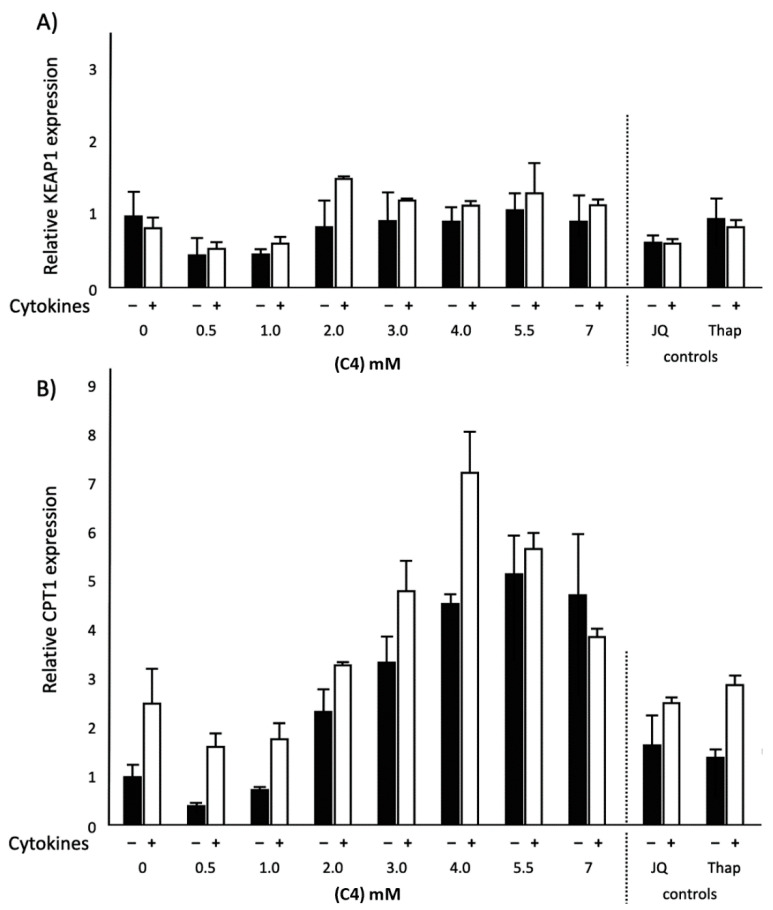
Relative kelch-like ECH-associated protein 1 (KEAP1) and carnitine palmitoyltransferase 1 (CPT1) mRNA expressions in HepG2 cells treated with different concentrations of C4, JQ1(+) (3 μM) or thapsigargin (Thap) (0.01 μM). (**A**) Increasing C4 concentrations showed a significant reduction in KEAP1 mRNA expression in the normal condition (*p* < 0.05). Increasing C4 concentrations showed a significant increase in KEAP1 mRNA expression in the inflammatory condition (*p* < 0.05) (**B**) Increasing C4 concentrations showed a significant increase in CPT1 mRNA expression in both normal and inflammatory conditions (*p* < 0.001). All results are presented as the mean, while error bars indicate standard deviations. Data were normalized against the expression observed in the control condition, which was arbitrarily set at 1. A quadratic polynomial regression was performed for C4 dose–response effects. Changes were considered significant when the regression coefficients were significantly different from zero (*p* < 0.05).

**Figure 3:**
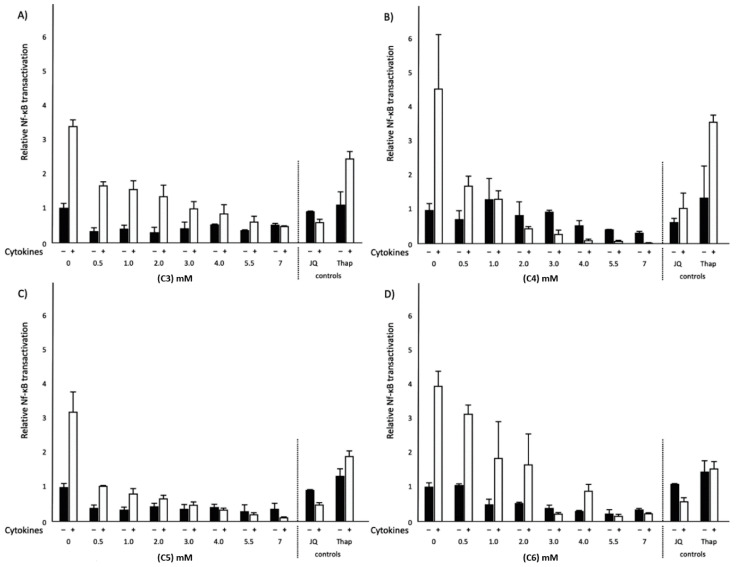
Relative nuclear factor kappa b (NF-κB) transactivation in HepG2 cells treated with different concentrations of SCFAs, JQ1(+) (3 μM) or thapsigargin (Thap) (0.01 μM). (**A**) Increasing C3 concentrations showed a significant decrease in NF-κB transactivation in the inflammatory condition (*p* < 0.05). (**B**) Increasing C4 concentrations showed a significant decrease in NF-κB transactivation in both normal and inflammatory conditions (*p* < 0.05). (**C**) Increasing C5 concentrations showed a significant decrease in NF-κB transactivation in the inflammatory condition (*p* < 0.05). (**D**) Increasing C6 concentrations showed a significant decrease in NF-κB transactivation in both normal and inflammatory conditions (*p* < 0.05). All results are presented as the mean, while error bars indicate standard deviations. Data were normalized against the transactivation observed in the control condition, which was arbitrarily set at 1. Linear regression for SCFA dose–response effects was performed except for C4, where a quadratic polynomial regression was performed to evaluate the dose–response effects. Changes were considered significant when the regression coefficients were significantly different from zero (*p* < 0.05).

To understand the possible mechanism underlying how C3, C4 and C5 rescued the reduced ApoA-I mRNA during inflammation, we first explored the effects of C4 on kelch-like ECH-associated protein 1 (KEAP1) and carnitine palmitoyltransferase 1 (CPT1) mRNA gene expressions and nuclear factor kappa b (NF-κB) transactivation. C4 dose-dependently increased KEAP1 mRNA expression (*p* < 0.05) under the inflammatory condition, while KEAP1 mRNA expression was reduced in the normal condition (*p* < 0.05). Furthermore, CPT1 mRNA expression dose-dependently increased (*p* < 0.001) after C4 treatment, both under normal and inflammatory conditions (Figure 2). When cells were transfected with the NF-κB reporter, both in normal as well as inflammatory conditions, C4 significantly (*p* < 0.05) decreased NF-κB transactivation (Figure 3). Moreover, an inverse correlation was found between CPT1 mRNA expression and NF-κB transactivation in inflamed HepG2 cells after C4 treatment (r = −0.733; *p* < 0.05). Finally, looking at the effects of the positive and negative controls, as expected, the bromodomain and extra-terminal (BET) inhibitor JQ1(+) significantly decreased KEAP1 gene expression (*p* < 0.05) both under inflammatory and normal conditions, whereas Thap did not affect KEAP1 gene expression. Furthermore, both JQ1(+) and Thap significantly (*p* < 0.05) increased CPT1 gene expression in both conditions (Figure 2).

### 2.3. Effects of Different SCFAs on NF-κB Transactivation and IL-8 Secretion in Normal and Inflammatory Conditions

**Figure 4:**
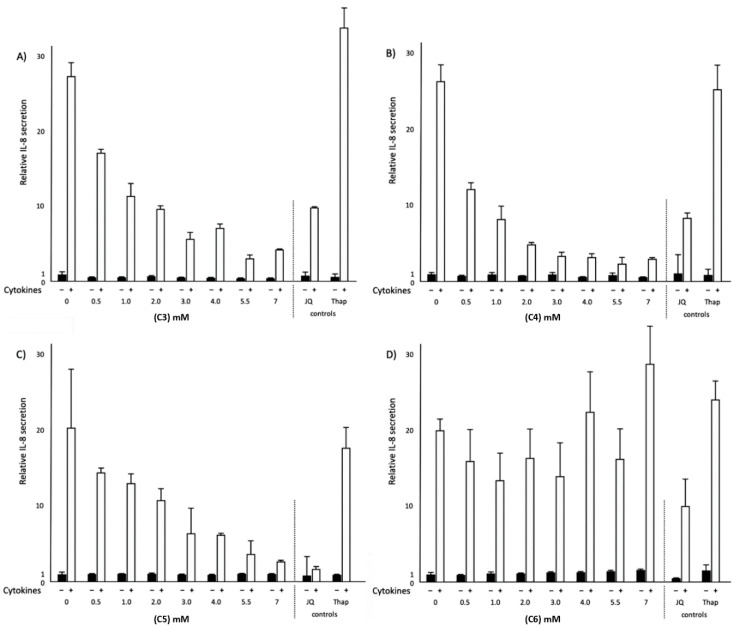
Relative interleukin 8 (IL-8) secretion in HepG2 cells treated with different concentrations of SCFAs, JQ1(+) (3 μM) or thapsigargin (Thap) (0.01 μM). (**A**) Increasing C3 concentrations showed a significant decrease in IL-8 secretion in the inflammatory condition (*p* < 0.05). (**B**) Increasing C4 concentrations showed a significant decrease in IL-8 secretion in the inflammatory condition (*p* < 0.05). (**C**) Increasing C5 concentrations showed a significant decrease in IL-8 secretion in the inflammatory condition (*p* < 0.05). (**D**) Increasing C6 concentrations did not show any significant effects on IL-8 secretion in either normal or inflammatory conditions. All results are presented as the mean, while error bars indicate standard deviations. Data were normalized against secretion observed in the control condition, which was arbitrarily set at 1. Linear regression for SCFAs dose–response effects was performed except for C4, where a quadratic polynomial regression was performed to evaluate the dose–response effects. Changes were considered significant when the regression coefficients were significantly different from zero (*p* < 0.05).

To extend the observed effects of C4 on NF-κB transactivation, the above-mentioned experiments for C4 were repeated with C3, C5 and C6. In the normal condition, not only C4 but also C6 significantly decreased NF-κB transactivation (*p* < 0.05), whereas C3 and C5 did not have any effects on NF-κB transactivation. In the inflammatory condition, all SCFAs significantly (*p* < 0.05) decreased NF-κB transactivation (Figure 3). Moreover, all SCFAs studied did not affect interleukin 8 (IL-8) secretion in normal conditions. On the other hand, in the inflammatory condition, C3, C4 and C5 significantly (*p* < 0.05) decreased IL-8 secretion (Figure 4). Interestingly, C6 did not lower IL-8 secretion even though C6 lowered NF-κB transactivation. JQ1(+) did not affect NF-κB transactivation, whereas Thap significantly (*p* < 0.05) increased NF-κB transactivation in both conditions (Figure 3). Finally, both JQ1(+) and Thap significantly (*p* < 0.05) further increased IL-8 secretion under the inflammatory condition, but these effects were not observed under the normal condition (Figure 4).

### 2.4. Effects of Different SCFAs on c-Jun and c-Fos mRNA Expression in Normal and Inflammatory Conditions

**Figure 5:**
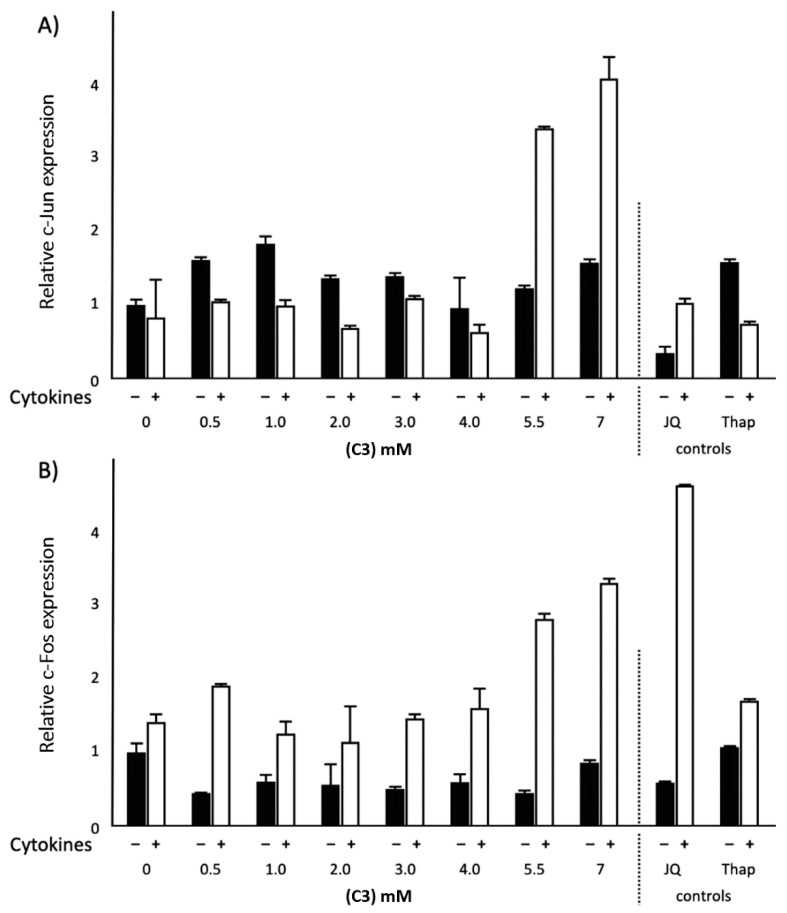
Relative c-Jun and c-Fos mRNA expressions in HepG2 cells treated with different concentrations of C3, JQ1(+) (3 μM) or thapsigargin (Thap) (0.01 μM). (**A**) Increasing C3 concentrations showed a significant increase in c-Jun mRNA expression in the inflammatory condition (*p* < 0.01). (**B**) Increasing C3 concentrations showed a significant increase in c-Fos mRNA expression in the inflammatory condition (*p* < 0.01). All results are presented as the mean, while error bars indicate standard deviations. Data were normalized against the expression observed in the control condition, which was arbitrarily set at 1. A linear regression analysis to evaluate C3 dose–response effects was performed. Changes were considered significant when the regression coefficients were significantly different from zero (*p* < 0.05).

To further explore the role of C3, C4 and C5 in rescuing ApoA-I transcription during inflammation, we also examined—besides NF-κB transactivation—the effects of SCFAs on activation of the activator protein (AP-1) pathway by analyzing potential changes in c-Fos and c-Jun mRNA expression. If the AP-1 pathway was involved, we would expect a reduction in AP-1 activation as translated into lower c-Fos and c-Jun expression. In normal conditions, all SCFAs (except C3) significantly (*p* < 0.01) increased c-Jun mRNA expression. In the inflammatory condition, all SCFAs increased (*p* < 0.01) c-Jun mRNA expression. Furthermore, all SCFAs did not change c-Fos mRNA expression in the normal condition, except C4, which significantly increased (*p* < 0.001) c-Fos mRNA expression. Moreover, all SCFAs increased (*p* < 0.01) c-Fos mRNA expression in the inflammatory condition, except C6, which significantly decreased (*p* < 0.05) c-Fos mRNA expression. Effects of C3 are shown in (Figure 5), whereas the effects of all SCFAs are shown in Supplementary Table S1.

## 3. Discussion

The SCFAs propionic acid (C3), butyric acid (C4), valeric acid (C5) and hexanoic acid (C6) are produced after the fermentation of dietary fibers and resistant starches by the microbiota in the cecum and colon [9]. An increasing number of functions and beneficial effects of SCFAs on human metabolism have been described [14]. Amongst others, SCFAs have anti-inflammatory effects and modulate different processes including cell proliferation, hormone secretion and immune responses [16]. SCFAs are either absorbed and utilized by gut epithelial cells or transported directly to the liver via the portal vein [10]. Despite different reports describing the beneficial effects of SCFAs in HepG2 cells, the effects of SCFAs on human liver cells under inflammatory conditions have not been studied. We recently described a favorable effect of SCFAs on ApoA-I mRNA transcription [11], but these effects were observed in normal (non-inflamed) HepG2 cells. Exposure to a cocktail of inflammatory cytokines of tumor necrosis factor alpha (TNF-α) and interleukin 1 beta (IL-1β) has already been shown to decrease ApoA-I mRNA levels in HepG2 cells [17]. Here we report the effects of SCFAs in HepG2 cells exposed to these cytokines, in which ApoA-I mRNA transcription was indeed lower due to the inflammatory response. This lower ApoA-I transcription in cytokine-exposed cells is in line with the known effects of acute phase responses on lipoprotein metabolism [18,19].

We observed that all four SCFAs studied here have anti-inflammatory effects as shown by a lower NF-κB transactivation, which in turn (except for C6) rescued the inflammation-induced reduced hepatic ApoA-I mRNA expression. This seems to be a logical response since the ApoA-I gene contains several NF-κB binding sites in the promoter region in hepatocytes [20,21]. The anti-inflammatory effects of C3, C4 and C5 were confirmed by a reduction in IL-8 secretion into the supernatant of the cells. However, C6 did not affect IL-8 secretion in the inflamed hepatic cells, which was remarkable since C6 did lower NF-κB transactivation just like the three other SCFAs.

These findings for these SCFAs were in line with previous studies which also evaluated the relation between SCFA exposure and inflammation in other cells types. For example, Qiao et al. [22] showed that C4 inhibited TNF-α, IL-6 and myeloperoxidase activity by preventing NF-κΒ transactivation in the liver cells of Sprague–Dawley rats. Another study showed that both C4 and C3 reduced IL-6 and IL-8 levels in human umbilical vein endothelial cells that were stimulated with lipopolysaccharide (LPS) and TNF-α [23]. Moreover, Usami and coworkers showed that C3 and C4 both reduced TNF-α production and downregulated NF-κB transactivation in peripheral blood mononuclear cells [24]. Here we extend these observations of C3 and C4 to C5 in HepG2 cells and link this interesting finding to the rescued expression of ApoA-I during inflammation.

To investigate the mechanisms underlying the rescued ApoA-I transcription by SCFA treatment in relation to the observed reduced NF-κB transactivation in more detail, changes in markers for BET inhibition and PPARα transactivation were analyzed. This analysis was conducted via testing BET and PPARα target gene expression (KEAP1 and CPT1, respectively). In both normal and inflammatory conditions, C4 treatment increased CPT1 mRNA transcription, whereas KEAP1 mRNA transcription was decreased in the normal condition only. Furthermore, we found a significant negative correlation between CPT1 mRNA expression and NF-κB transactivation associated with C4 treatment in the inflammatory condition. Consequently, this finding suggests the ability of PPARα to reduce inflammation by inhibiting NF-κB transactivation. All together, these results of C4 suggested that PPARα transactivation might be involved in the effects of SCFAs on ApoA-I expression in normal and inflammatory conditions. This is also in agreement with our previous study in which we found a clear role for PPARα transactivation on ApoA-I mRNA transcription in non-inflamed HepG2 cells [15]. Moreover, it seems that BET inhibition does not contribute to the effects of SCFAs on ApoA-I during inflammatory conditions, since KEAP1 was not reduced in inflamed HepG2 cells.

We observed variations in the ability of SCFAs to increase ApoA-I expression or to reduce IL-8 secretion. In our previous study in non-inflamed conditions [11], we have shown that C6 was the weakest inducer of ApoA-I mRNA expression as compared to the other SCFAs (C3, C4 and C5). Again, in the present study, we found that C6 was the only SCFA that was unable to rescue ApoA-I transcription in the inflammatory condition. Moreover, although C6 reduced NF-κB transactivation just like the other SCFAs, we found that C6 did not inhibit IL-8 secretion like the other SCFAs. This outcome regarding the effects of C6 suggests that the anti-inflammatory effects of SCFAs related to ApoA-I might not be linked only to a blunted NF-κB transactivation but could additionally also be ascribed to other (inflammatory) pathways. Since the observed reduction in IL-8 was associated with increased ApoA-I expression after C3, C4 and C5 treatment, while C6 did not lower IL-8 expression and also did not elevate ApoA-I expression, we speculated that the ApoA-I rescue effects of C3, C4 and C5 could also be the result of another modulating anti-inflammatory signaling pathway underlying IL-8 secretion. The promoter region of the IL-8 gene contains not only functional binding sites for NF-κB, but also for AP-1 and CCAAT/enhancer binding protein β (C/EBP-β) [25]. It has been described that PPARα, which is known as a regulatory factor of ApoA-I [15], is also linked to the AP-1 pathway [26]. PPARα transactivation had an inhibitory effect on both NF-κB and AP-1, which led to the inhibition of inflammation [26]. Although an earlier study showed a significant correlation between SCFA exposure and the regulation of the AP-1 signaling pathway in intestinal cells [27], the relationship between SCFAs and the AP-1 pathway in inflamed liver cells has not been studied before. Consequently, we decided to investigate the anti-inflammatory effects of SCFAs in relation to the AP-1 signaling pathway as an additional candidate besides lowering NF-κB pathway activity. Therefore, the mRNA expression of c-Jun and c-Fos, both target genes of the AP-1 pathway, were evaluated in both normal and inflamed HepG2 cells. Unfortunately, all SCFAs, again except C6, did not inhibit the AP-1 signaling pathway in the inflammatory condition. This means that inhibition of the AP-1 pathway was probably not involved in the anti-inflammatory effects of those SCFAs that were able to elevate ApoA-I mRNA expression. However, since the transcriptional activity of c-Jun is regulated mainly post-transcriptionally via phosphorylation, other types of experiments need to be performed to confirm the absence of AP-1 involvement. Altogether, our findings regarding the relationship between exposure to SCFAs and NF-κB or AP-1 pathway activation allow us to speculate that maybe the third candidate regulator C/EBP-β with a binding site in the IL-8 promoter might be a potential mediator involved in the effects of SCFAs during inflammation. Indeed, Bai and coworkers have shown that overexpression of C/EBP-β increased the expression of cytokines such as IL-8 [28]. Furthermore, the ApoA-I promoter has a C/EBP binding site, which indicates that C/EBP-β might be involved in ApoA-I production [29]. As a result, we suggest in future experiments to investigate the role of SCFAs in rescuing the inflammation-induced reduction in ApoA-I expression by evaluating their effects on the C/EBP- β signaling pathway.

For now, we conclude that the SCFAs propionic acid, butyric acid and valeric acid can reduce NF-κB mediated pro-inflammatory responses, probably mediated via PPARα transactivation, which translates into a rescue of ApoA-I transcription in inflamed HepG2 cells. This anti-inflammatory effect of C3, C4 and C5 was confirmed by the inhibition of both NF-κB transactivation and IL-8 secretion.

## 4. Material and Methods

### 4.1. Materials

Human hepatocellular liver carcinoma cells (HepG2) were kindly provided by Sten Braesch-Andersen (Mabtech, Nacka Strand, Sweden). Cell culture flasks and plates were obtained from Corning (Corning, NY, USA). Minimum Essential Medium (MEM), sodium pyruvate, non-essential amino acids (NEAA), penicillin and streptomycin were all obtained from Thermo Fisher Scientific (Bleiswijk, Netherlands). Fetal bovine serum (FBS) was purchased from PAA (Toronto, ON, Canada). Propionic acid (C3), butyric acid (C4), valeric acid (C5) and hexanoic acid (C6) were bought from Sigma (Uithoorn, Netherlands). JQ1(+) was obtained from Bio-Techne R&D (Minneapolis, MN, USA). Tumor necrosis factor-alpha (TNF-α), interleukin-1 beta (IL-1β), thapsigargin (Thap), dimethyl sulfoxide (DMSO) and Tri-reagent were purchased from Sigma (Uithoorn, Netherlands).

### 4.2. Cell Culture and SCFA Treatment

HepG2 cells were cultured at 37 °C in a humidified atmosphere of 5% carbon dioxide (CO_2_) in MEM containing 10% heat inactivated FBS, 1% sodium pyruvate, 1% NEAA and 1% of penicillin–streptomycin mixture. For all experiments, cells were seeded in a 24-well plate at a density of 200,000 cells per well. Cell viability was inspected daily by microscope and when cells reached a density of 80–90%, they were incubated for 48 h in the medium (MEM without FBS) plus a concentration range of 0–7 mM SCFAs (C3, C4, C5 or C6) or 3µM JQ1(+) with or without a cytokine cocktail (TNF-α 100 ng/mL and IL-1β 5 ng/mL). A positive control JQ1(+), a BET inhibitor, was included in each experiment to ensure that cells were responsive and produced sufficient amounts of ApoA-I mRNA. Thapsigargin (Thap), an endoplasmic reticulum (ER)stress inducer, was used as a negative control. All SCFAs, JQ1(+) and Thap were dissolved in dimethyl sulfoxide (DMSO, cell culture tested) and effects were expressed relative to those of the carrier control (DMSO only). The final DMSO concentration was always 0.2%. Culture medium was collected for the analysis of IL-8 concentrations and cells were harvested for the analysis of mRNA expression after lysing with Tri-reagent. Both culture medium and lysed cells were snap frozen in liquid nitrogen and stored at −80 °C until further analysis.

### 4.3. Quantification of Gene mRNA Transcription

To evaluate effects of SCFAs on mRNA expression levels of ApoA-I, KEAP1, CPT1, c-Jun and c-Fos, total RNA was isolated from HepG2 cells using Tri-reagent according to the manufacturer’s instructions. The RNeasy Mini Kit (Qiagen, Hilden, Germany) was used for RNA purification. For cDNA synthesis, 350 ng of total RNA was reverse-transcribed using RNAse inhibitor, dNTPs, random hexamers, moloney murine leukemia virus (MMLV) reverse trans, Dithiothreitol (DTT) and 5x reverse transcriptase (RT) buffer (Thermo Fisher Scientific, Bleiswijk, Netherlands). The resulting cDNA was used for real-time quantitative PCR using TaqMan Gene Expression Assays using cyclophilin A (Hs99999904) as a housekeeping control. To quantify ApoA-I, KEAP1, CPT1, c-Jun and c-Fos, the TaqMan gene expression assays Hs00163641, Hs00202227, Hs00912671, Hs00277190 and Hs00170630 were used. Values are presented as relative gene expressions based on the cycle threshold (Ct) values, normalized for the internal control cyclophilin A, and compared to the control conditions.

### 4.4. Luciferase Assay

To investigate effects of SCFAs on NF-κB transactivation, HepG2 cells were transfected with X-treme gene 9 DNA transfection reagent (Sigma, Uithoorn, Netherlands) and the plasmids pcDNA3.1, pGL3 and NF-κB pGL3 following the manufacturer’s instructions. The pcDNA3.1 (empty vector) and the empty pGL3 luciferase reporter (without NF-κB elements) were used as a negative control. Following transfection and 48 h SCFA treatment with or without the cytokine cocktail, cells were lysed in luciferase lysis buffer (Promega, Madison, WI, USA) and measured for luciferase activity, reflecting NF-κB transactivation, using a GloMax^®^ 96 Microplate luminometer, according to the manufacturer’s manual (Promega, Madison, WI, USA).

### 4.5. Quantification of IL-8 Secretion Levels in the Culture Medium

To investigate IL-8 secretion by HepG2 cells, IL-8 protein concentrations in culture medium were measured by an enzyme-linked immunoassay (ELISA; Hycult Biotechnology, Uden, The Netherlands) following the manufacturer’s instructions.

### 4.6. Statistical Analysis

All independent dose–response experiments with the SCFAs were performed in duplicate and each experiment was repeated three times. Six biological (12 technical) replicates were performed for every single SCFA dose. Regression analysis was used to examine the relationships between SCFAs and the parameters. First, we modeled via quadratic polynomial regression, which fits with a parabolic relationship as seen for C4. In case this did not reach significance, a linear relationship (i.e., as seen for C3 and C5) was examined. Where mentioned, Spearman correlations were calculated. The regression coefficients and Spearman correlation coefficients were considered to be statistically significant when different from zero at *p* < 0.05. Effects of the positive JQ1(+) and negative (Thap) controls were statistically evaluated by Mann–Whitney U test, in which a *p*-value < 0.05 was considered statistically significant. All statistical analyses were performed using SPSS v.25 (IBM Corp., Armonk, NY, USA).

## Supplementary Materials

The following are available online at https://www.mdpi.com/1422-0067/21/14/5088/s1, Supplementary Table S1.
